# Awareness of Traumatic Dental Injuries and Impact of Educational Intervention Among Croatian Soccer Coaches and Players

**DOI:** 10.3390/dj13030121

**Published:** 2025-03-10

**Authors:** Dina Bursać, Lovro Marinović, Marta Horvat, Kristina Goršeta

**Affiliations:** 1 University Hospital Center Zagreb, 10 000 Zagreb, Croatia; dina.cigra@gmail.com; 2 University Hospital Merkur, 10 000 Zagreb, Croatia; 3 Health Center Zagreb-West, 10 000 Zagreb, Croatia; 4 Department of Pediatric and Preventive Dentistry, University of Zagreb School of Dental Medicine, 10 000 Zagreb, Croatia

**Keywords:** dental trauma, prevention, mouthguard

## Abstract

**Background/Objectives**: This study aimed to assess the knowledge of dental injuries in both soccer coaches and players, as well as the impact of educational intervention on coaches and the prevalence of traumatic dental injuries and mouthguard usage among soccer players. **Methods**: The study involved 94 male soccer players (median age 18, IQR: 12–19) and 39 coaches. Data were collected through questionnaires covering age, gender, years of experience, injury history, mouthguard use, and knowledge of tooth rescue kits and tooth repositioning. **Results**: Of the players, 34 (36.2%) experienced orofacial injuries during soccer, while only 7 (7.4%) used mouthguards. Reasons for not using mouthguards included discomfort (26.1%), belief they are unnecessary (26.1%), and difficulty purchasing them (8.0%). Among coaches, 24 (61.5%) reported handling dental injuries during training, including crown fractures (41.7%), tooth avulsions (33.3%), and tooth luxations (25.0%). Only four (10.3%) coaches personally used mouthguards. **Conclusions**: The study identified a significant lack of knowledge about dental injuries and limited use of mouthguards among Croatian soccer players. Coaches, while influential in athlete development, should also focus on injury prevention, including dental trauma.

## 1. Introduction

Soccer is widely considered one of the most popular sports globally, drawing millions of players from all corners of the globe. As a dynamic team sport, soccer involves much more than simply kicking a ball to score a goal. Players engage in a range of actions, including passing, tackling, evading tackles, catching and kicking. The fast-paced nature of the game often leads to both intentional or unintentional physical contact, making soccer inherently risky. Consequently, players are exposed to a heightened likelihood of craniofacial injuries, including teeth injuries. The research indicates that the prevalence of dental trauma among soccer players ranges from 11% to 21% [1,2,3]. Despite these risks, the use of mouthguards is not a mandatory part of protective equipment in soccer; its use remains optional and dependent on individual player preferences.

Traumatic dental injuries in soccer often occur during training sessions or matches, where physical contact is frequent and sometimes intense. When these injuries happen, coaches or medical personnel present on the field are typically the first to respond to the injured player. Educating coaches on emergency procedures for dental trauma is important, as it enables them to take appropriate actions that can significantly improve the prognosis and recovery outcomes for the injured player. Coaches should also be well-versed in preventive methods and encourage players to use mouthguards to protect their teeth and faces from injury [4,5,6,7].

Previous research has highlighted a concerning gap in knowledge among school teachers [8,9,10] and sports coaches [11,12] concerning the prevention of dental trauma and appropriate emergency procedures following dental luxation. The initial hours following a dental trauma are critical for preserving the vitality of the affected teeth. During this time, it is crucial for the injured player to reach a dental office for professional treatment [13].

Despite the well-documented risk associated with sports, many athletes remain unaware of the potential risk for dental trauma. Research carried out by Levin et al., which involved 943 athletes, revealed that more than 70% were unaware of the risk of dental injuries that could occur on the sports field. Additionally, a significant number were not familiar with the protective benefits of mouthguards [14]. This lack of awareness is particularly pronounced among amateur players, who often underestimated their risk of injury and were less likely to use mouthguards [15]. Multiple studies have shown that wearing mouthguards can significantly reduce the incidence of craniofacial and dental trauma in athletes [16,17,18]. When coaches understand the importance of mouthguards, they are more likely to encourage their use among players, helping to mitigate the risk of dental injuries. Educational initiatives aimed at improving coaches’ knowledge and attitudes toward injury prevention could also improve the management of dental injuries when they do occur, leading to better treatment outcomes for players. The need for effective educational programs is evident in ensuring that players, especially at the amateur level, take proactive steps to protect themselves from dental trauma [14,15,16,17,18].

The purpose of this study was to assess the knowledge of Croatian soccer coaches regarding dental and facial injuries as well as their awareness regarding injury prevention and emergency procedures following dental trauma, both before and after an intervention in the form of an educational seminar. Another goal was to estimate the prevalence of traumatic dental injuries among soccer players and assess their habits concerning mouthguard usage. The findings of this study could serve as a basis for future educational programs and policies aimed at protecting athletes from dental trauma.

## 2. Materials and Methods

This cross-sectional study was approved by the University of Zagreb, School of Dental Medicine Ethics Committee. The study was conducted among licensed soccer coaches and players registered by the Croatian Football Federation. Participation in this study was entirely voluntary, with participants assured of their anonymity. They were informed of their right to withdraw at any time without any consequences. Informed consent was obtained directly from adult participants, and consent was obtained from children’s parents or guardians.

### 2.1. Participant Recruitment

#### 2.1.1. Coach Recruitment

A total of 50 soccer coaches were initially contacted to participate in the study. These coaches were attending a re-licensing seminar organized by the Croatian Football Federation. Since all Croatian coaches are required to periodically attend these seminars, the sample was considered representative of the coaching population.

Eligibility criteria for coaches are as follows:

Minimum one year of experience working with youth soccer players;

Actively leading a team during the study period.

Both professional coaches (primary occupation) and amateur coaches (voluntary or secondary occupation) were included.

Out of the 50 coaches approached, 39 agreed to participate (78% acceptance rate), while 11 declined due to time constraints or lack of interest.

#### 2.1.2. Player Recruitment

Soccer players were recruited during the national championship for amateur players in Croatia. This annual event gathers players of different ages from across the country.

To facilitate participation, researchers set up a supervised area where players could complete the survey without distractions. Participation was voluntary, and a total of 94 soccer players expressed interest and were included in the study.

Data collection was carried out using questionnaires specifically designed for this study, which were based on the existing literature and validated questionnaires from previous studies [19,20,21,22,23]. Two distinct questionnaires were used: one for soccer players and one for soccer coaches. The questionnaires for both players and coaches included questions on demographic information (age, gender, years of experience in soccer), firsthand and secondhand experiences of craniofacial injuries, and the use of mouthguards. Additionally, they contained questions assessing knowledge and attitudes towards different types of mouthguards, tooth rescue kits, and tooth repositioning techniques. The coaches completed questionnaires before and after attending educational lectures on craniofacial injuries.

### 2.2. Educational Intervention for Coaches on Craniofacial Injuries

#### 2.2.1. Intervention Format

A structured educational session was delivered to small groups of coaches in the form of an oral presentation. The session aimed to enhance their knowledge and preparedness regarding craniofacial injuries in sports.

#### 2.2.2. Content of the Session

- The presentation covered key topics, including the following:
- The risks and consequences of dental injuries;
- The importance of immediate and appropriate responses;
- The benefits of using mouthguards for injury prevention.

#### 2.2.3. Assessment of Knowledge and Attitudes

To evaluate the effectiveness of the intervention, pre- and post-session surveys were conducted to measure changes in the coaches’ knowledge and attitudes.

#### 2.2.4. Duration and Delivery

The session lasted 35 min and was conducted by a doctor of dental medicine, ensuring expert-driven and authoritative content. The brief yet focused format allowed for an engaging learning experience without overwhelming the participants.

Categorical variables were expressed as absolute numbers and percentages, while continuous variables were reported as means with standard deviations (SD) for normally distributed data and as medians with interquartile ranges (IQR) for non-normally distributed data. The Shapiro–Wilk test was employed to assess the normality of distribution for continuous variables. Comparisons of categorical variables were made using Fisher’s exact test, and the exact McNemar’s test was used to evaluate changes in participants’ knowledge before and after the seminar. A statistical significance level was set at α = 0.05. All statistical analysis was conducted using IBM SPSS Statistics for Windows (ver. 26.0., IBM Corp, Armonk, NY, USA).

## 3. Results

The study included 94 soccer players, all of whom were men. The median age of players was 18 (IQR: 12–19) years. A total of 34 (36.2%) players reported experiencing orofacial injuries during soccer-related activities. The distribution of injury types and comparison across age groups is shown in Table 1. Players also answered a set of questions concerning their knowledge and attitudes about dental trauma and mouthguards, with the responses summarized in Table 2.

Among the players, only seven (7.4%) reported using mouthguards, and all of them used premade (“stock”) mouthguards. The most commonly cited reasons given for not wearing a mouthguard included discomfort while wearing one (26.1%), the belief that mouthguards are not useful (26.1%), and difficulty purchasing a mouthguard (8.0%).

The study also included a total of 39 coaches, out of whom 38 (97.4%) were men. The mean age of the coaches was 43.5 years (SD ±10.70), and they had an average of 15.9 years (SD ± 8.30) of coaching experience. A significant portion of the coaches, 24 (61.5%), had encountered athletes with dental injuries during training sessions. The most common types of dental injuries reported by the coaches were crown fractures (10 cases, 41.7%), tooth avulsions (8 cases, 33.3%), and tooth luxations (6 cases, 25.0%).

Interestingly, only four coaches (10.3%) reported using mouthguards themselves. The study’s participants included 13 professional coaches (33.3%) and 26 amateur coaches (66.6%). The differences in attitudes and educational backgrounds between professional and amateur coaches are outlined in Table 3. Additionally, the results of the coaches’ knowledge of dental injuries before and after attending an educational seminar are presented in Table 4.

## 4. Discussion

Dental and craniofacial injuries are prevalent in sports, with up to one-third of craniofacial injuries attributed to athletic activities. In the hierarchy of dentofacial injury prevalence in sports, rugby has the highest incidence, followed by basketball, handball, field hockey, and soccer. Despite the high incidence of these injuries in soccer, there is a notable gap in the knowledge and awareness among coaches, especially concerning the importance and benefits of mouthguards. Although soccer is not classified as a full-contact sport, it poses a significant risk for dental and craniofacial injuries due to its dynamic and sometimes aggressive nature [24]. The lack of mandatory protective devices and the physical interactions among players contribute to this risk. This study revealed a significant prevalence of dental injuries among soccer players, with older players experiencing more injuries. The most prevalent type of injury was crown fracture, emphasizing the need for protective measures in this sport.

Lip lacerations have consistently been the most commonly reported orofacial injury in previous studies. However, their consequences are generally less severe compared to dental trauma. Dental trauma often has more severe physical, economic, and psychosocial consequences. In soccer, dental injuries often occur due to blows to the face from hands or elbows during collisions, frequently stemming from the game’s fast pace and physical nature. Head-to-head impacts and close-range ball strikes are also common causes of such injuries [25].

In this study, dental injuries were more common in older players, with crown fractures being the most prevalent type (41.7%). These findings are consistent with other studies on this topic [2]. When compared to dental injury prevalence in contact sports such as taekwondo [26], judo, or wrestling [27], traumatic dental injuries are as common or even more common in soccer than in contact sports [28]. This paradox can be attributed to the mandatory use of mouthguards in contact sports and the greater awareness of dental trauma among players and coaches [26].

Mouthguards are available in three types: prefabricated (stock), mouth-formed, and custom-made, with the latter providing the highest level of protection and comfort. Custom-made mouthguards have additional advantages, including minimal interference with breathing and speech, a lower risk of causing nausea, and increased durability. Their primary role in sports is to prevent or mitigate injuries to the teeth, gingival tissues, lips, and jaws. They function by distributing the force of impact, thereby reducing the energy transmitted to the dentition.

In our study, only 7.4% of surveyed soccer players reported that they wear a mouthguard during sports activities, and all of them used prefabricated mouthguards, which provide the poorest level of protection and comfort. The primary reasons for not wearing a mouthguard were discomfort and lack of fit, perceived uselessness in preventing injury, and difficulty purchasing one. The average level of knowledge among the surveyed players about dental trauma and mouthguard use was inadequate, mirroring findings from studies on amateur and professional football players in Kuwait and the Netherlands [3,29]. Although players typically spend more time training than playing in games, the majority of injuries occur during matches, as the intensity of competition rises, leading to a higher risk of injury and trauma.

The results of the coaches’ survey revealed a significant difference between amateur and professional coaches. Most amateur coaches (96.2%) had never attended a first aid course, compared to 8.3% of professional coaches. The study showed that more professional coaches (76.9%) had received educational materials compared to amateur coaches (61.5%). Professional coaches reported receiving educational materials more often than amateur coaches. Amateur coaches had less access to educational materials on dental trauma than professional coaches (61.5 and 76.9%, respectively) and were, on average, less satisfied with their level of knowledge on dental injuries than professional coaches (38.5 and 69.2%, respectively). However, amateur coaches reported encountering soccer-related dental trauma more frequently than their professional counterparts. This discrepancy is consistent with studies by van Vliet et al. and Sepet et al. [6,30].

The findings of this study indicate a disparity in knowledge of emergency dental injury procedures among coaches, depending on the league in which they are involved. A comparable study conducted in Brazil yielded similar results, revealing that coaches in the professional soccer category demonstrated a higher level of awareness regarding dental trauma prevention than their counterparts in amateur soccer. This difference is likely a reflection of the technical resources available to professional soccer clubs, which typically offer more extensive injury prevention programs and insurance coverage [31].

The study also examined the effects of an educational seminar on coaches’ knowledge and attitudes. Before the seminar, approximately 59%of the coaches could distinguish different types of mouthguards, and 72 and 79% of coaches knew about tooth replantation kits and the possibility of tooth replantation, respectively. Post-seminar, coaches showed marked improvement in knowledge on these topics, but there remains significant room for improvement. This points to a need to make the seminars simpler and more engaging. Furthermore, better educational outcomes could be achieved by using additional methods such as workshops with smaller groups, posters, or quizzes. A similar study was performed by Young et al., in which a poster on the management of dental trauma proved to be effective in increasing the levels of knowledge in Hong Kong Teachers [19].

Despite the seminar’s positive impact on knowledge, coaches’ attitudes toward mouthguard use shifted negatively. This indicates a need to address preconceived notions about mouthguard use in soccer. Educating coaches about the benefits and types of mouthguards and the long-term consequences of dental injuries is crucial. In fact, soccer players are among the groups most at risk for craniofacial injury, as is shown in the results of our survey and the works of Kasum et al. [2].

One of the key strengths of this study was the comprehensive sampling process, which included both amateur and professional coaches from across the entire country. To effectively measure the long-term impact of educational interventions, follow-up data should be collected periodically, such as after one month and one year.

## 5. Conclusions

Both players and coaches exhibited limited knowledge regarding dental trauma management, including tooth replantation and the use of rescue kits. Although an educational seminar improved coaches’ knowledge, it did not positively shift attitudes toward recommending mouthguards. These results emphasize the urgent need for more engaging and comprehensive educational programs to enhance dental injury prevention and emergency response strategies within the soccer community.

## Figures and Tables

**Table 1:** Distribution of orofacial injuries by type and age group among Croatian soccer players.

Group of Athletes		Type of Injury				
		Tooth Injury	Tooth and Soft Tissue Injury	Jaw Fracture	Total	Fisher’s Exact Test
		N	N (%)	N (%)	N (%)	
Age group	Minors (8–17 y)	45	3 (6.7)	0	8 (17.8)	*p* < 0.001
	Seniors (18–28 y)	49	7 (14.3)	1 (1.1)	26 (53.1)	

**Table 2:** Knowledge and attitudes towards dental trauma and mouthguard usage among Croatian soccer players.

Question	Number of Answers (%)	
	YES	NO
Do you know it is possible to replant an avulsed tooth?	51 (54.3)	43 (45.7)
Do you know about a tooth rescue kit?	23 (24.5)	71 (75.5)
Do you think that wearing a mouthguard could be effective in your sports activity?	46 (48.9)	48 (51.1)
Do you use a mouthguard during sports activities?	7 (7.4)	87 (92.6)
Would you like to know more about mouthguards?	21 (22.3)	73 (77.7)

**Table 3:** A comparative analysis of dental trauma knowledge, experience, and first aid training among amateur and professional soccer coaches.

	Professional Coach	Amateur Coach	
Question	N = 13 (13.3)	N = 26 (66.6)	Fisher’s Exact Test
Has previously attended a first aid course			
Yes	11 (84.6)	1 (3.8)	*p* < 0.001
No	2 (8.3)	25 (96.2)	
Has experienced a case of an athlete’s dental injury			
Yes	7 (53.8)	17 (65.4)	*p* = 0.508
No	6 (46.2)	9 (34.6)	
Has received educational materials about dental trauma before			
Yes	10 (76.9)	16 (61.5)	*p* = 0.477
No	3 (23.1)	10 (38.5)	
Is satisfied with their knowledge on dental injuries			
Yes	9 (69.2)	10 (38.5)	*p* = 0.096
No	4 (30.8)	16 (61.5)	

**Table 4:** Effect of educational intervention on soccer coaches’ knowledge and attitudes towards dental trauma management and mouthguard use.

	Before the Lecture	After the Lecture	
Question	N = 39	N = 39	McNemar’s Test
Differentiates mouthguard types			
Yes	12 (30.7)	23 (58.9)	*p* = 0.027
No	27 (69.2)	13 (41.0)	
Knows about tooth preservation kit		0	
Yes	8 (20.5)	28 (71.8)	*p* < 0.001
No	31 (79.5)	11 (28.2)	
Knows of tooth replantation			
Yes	8 (20.5)	28 (71.8)	*p* < 0.001
No	31 (79.5)	11 (28.2)	
Knows of tooth replantation			
Yes	10 (25.6)	31 (79.5)	*p* = 0.019
No	29 (74.4)	8 (20.5)	
Would recommend a mouthguard			
Yes	35 (89.7)	29 (74.4)	*p* = 0.180
No	4 (10.3)	10 (25.6)	

## Data Availability

The original contributions presented in this study are included in the article. Further inquiries can be directed to the corresponding author.
